# Timing of endoscopy in patients with upper gastrointestinal bleeding

**DOI:** 10.1038/s41598-022-10897-3

**Published:** 2022-04-27

**Authors:** Jeemyoung Kim, Eun Jeong Gong, Myeongsook Seo, Jong Kyu Park, Sang Jin Lee, Koon Hee Han, Young Don Kim, Woo Jin Jeong, Gab Jin Cheon, Hyun Il Seo

**Affiliations:** Department of Internal Medicine, Gangneung Asan Hospital, University of Ulsan College of Medicine, Gangneung, Korea

**Keywords:** Gastroenterology, Medical research, Signs and symptoms

## Abstract

The optimal timing of endoscopy in patients with acute upper gastrointestinal bleeding (UGIB) remains controversial. In this study, we investigated the clinical outcomes of urgent endoscopy in patients with UGIB compared with elective endoscopy. From January 2016 to December 2018, consecutive patients who visited the emergency department and underwent endoscopy for clinical manifestations of acute UGIB, including variceal bleeding, were eligible. Urgent endoscopy (within 6 h) and elective endoscopy (after 6 h) were defined as the time taken to perform endoscopy after presentation to the emergency department. The primary outcome was mortality rate within 30 days. A total of 572 patients were included in the analysis. Urgent endoscopy was performed in 490 patients (85.7%). The 30-day mortality rate did not differ between the urgent and elective endoscopy groups (5.3% and 6.1%, *p* = 0.791). There was no difference regarding the recurrent bleeding rate, total amount of transfusion, or length of hospital between the groups. In multivariate analysis, age and the amount of transfusion were associated with mortality. Urgent endoscopy was not associated with a lower 30-day mortality rate compared with elective endoscopy in patients with acute UGIB.

## Introduction

Acute upper gastrointestinal bleeding (UGIB) is one of the most common gastrointestinal emergencies. Despite remarkable advancements in endoscopic treatments and substantial efforts in reducing mortality, the overall in-hospital mortality rate associated with UGIB is still estimated to be 10%^1^. Endoscopic examination plays a pivotal role in both the diagnosis and treatment of UGIB^2^. Current guidelines recommend performing endoscopy within 24 h of patient presentation, while emphasizing hemodynamic stabilization before the procedure^3–6^. However, the optimal timing of endoscopy within those 24 h and the benefit of earlier endoscopy remains controversial.

Several studies have investigated the clinical impact of urgent (within 6 h of presentation) or early (within 12 h) endoscopy on mortality in patients with UGIB. Some studies showed no significant difference in mortality rate between urgent and elective endoscopy groups among high-risk patients with acute UGIB^7,8^. In contrast, others found that urgent endoscopy was associated with a lower mortality rate in high-risk patients with acute non-variceal UGIB^9,10^.

Most of the previous studies included highly selected patients with non-variceal UGIB or those at a high risk^7–12^. However, given that the definitive diagnosis is made after endoscopic examination, these studies may not reflect real-world clinical practice. Furthermore, it is often challenging to predict the cause of bleeding and to identify patients at high risk who require intensive care based on their symptoms and the information obtained in the emergency room^13^. In this study, we investigated the clinical outcomes of urgent endoscopy in patients with UGIB compared with elective endoscopy.

## Methods

**Figure 1:**
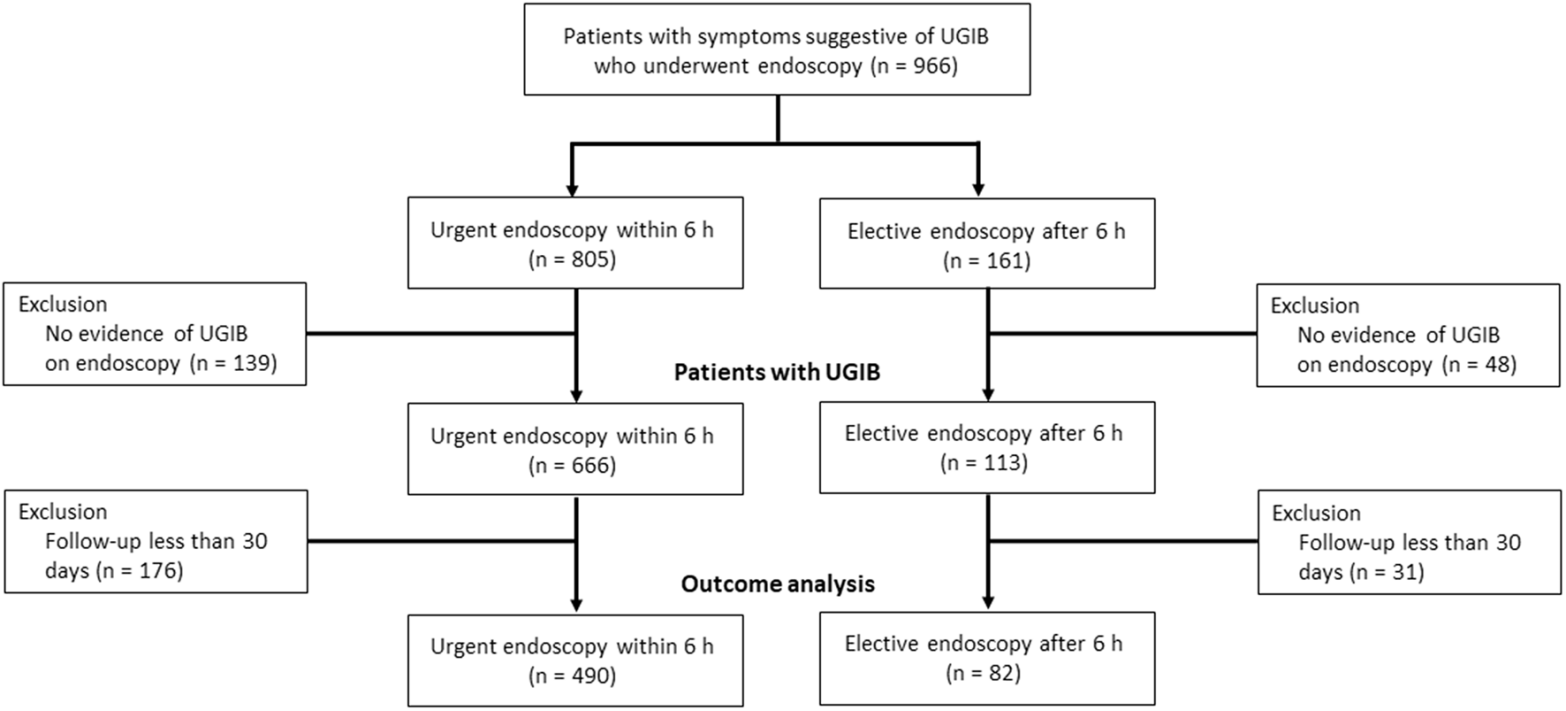
Flowchart of the study. *UGIB* upper gastrointestinal bleeding.

From January 2016 to December 2018, a total of 966 consecutive patients visited the emergency department with symptoms suggestive of UGIB and underwent endoscopy. Of these, 187 patients with no evidence of UGIB on endoscopy and 207 patients with a follow-up period of fewer than 30 days were excluded. Finally, a total of 572 patients with UGIB were included in the analysis (Fig. 1).

Patient-related factors (age, sex, comorbidities, previous history of peptic ulcer, and medication history), clinical parameters (presenting symptoms, systolic blood pressure, heart rate, laboratory findings, amount of transfused red blood cell (RBC), and duration of hospital stay), and procedure-related factors (time to endoscopy, endoscopic diagnosis, and kind of hemostasis) were evaluated using medical records. The Glasgow-Blatchford score (GBS) was calculated, using systolic blood pressure, heart rate, hemoglobin, blood urea nitrogen, the presence of melena or syncope, and the presence of hepatic disease or cardiac disease^14^.

Patients were divided into two groups according to the timing of endoscopy which was defined as the time taken to perform endoscopy after presentation: the urgent endoscopy group (within 6 h, n = 490, 85.7%), and the elective endoscopy group (after 6 h, n = 82, 14.3%). Endoscopic treatment was performed using techniques such as thermocoagulation, hemoclip, injection, band ligation, and a combination of two or more techniques. The type of treatment was determined by the endoscopist. The primary outcome was the mortality rate within 30 days. Secondary outcomes included rebleeding rate within 30 days, median duration of hospital stay, and median amount of RBC transfusion.

### Statistical analysis

Continuous variables are shown as median (range), and categorical variables are shown as number (percentage). Differences in baseline characteristics were tested by the chi-square test, Fisher`s exact test, *t*-test, or Mann–Whitney *U*-test, as appropriate. A logistic regression model was used to identify factors associated with mortality, and odds ratio (OR) and 95% confidence interval (CI) were estimated. All statistical analyses were performed by using SPSS v21.0 (SPSS Inc., Chicago, USA).

### Ethics information

Approval for accessing patient information was granted from the Institutional Review Board of Gangneung Asan Hospital (2020-03-009). As this retrospective data collection was considered anonymized by the Ethics committee, the need for patient consent was waived by the Institutional Review Board of Gangneung Asan Hospital. The study was conducted according to good clinical and scientific practices and following the ethical principles of the Declaration of Helsinki.

## Results

### Characteristics of the study population

The baseline characteristics of the study population are summarized in Table 1. The median age of the 572 patients was 63 years (range, 19–95 years) and 64.0% were male. More than 80% of the patients had comorbidities, including diabetes mellitus, cerebrovascular accident, chronic kidney disease, or liver cirrhosis. The proportion of patients who were taking antithrombotic agents at the time of admission was 23.8%, and 25 patients (4.4%) were on dual antiplatelet therapy.

**Table 1:** Characteristics of the study population.

	Total (N = 572)	Urgent (n = 490)	Elective (n = 82)	*P* value
Age, years	63 (19–95)	63 (21–95)	62 (19–93)	0.879
Male	366 (64.0)	312 (63.7)	54 (65.9)	0.712
Comorbidities	461 (80.6)	394 (80.4)	67 (81.7)	0.881
Diabetes mellitus	160 (28.0)	135 (27.6)	25 (30.5)	
Cerebrovascular accident	63 (11.0)	58 (11.8)	5 (6.1)	
Vascular disease	41 (7.2)	34 (6.9)	7 (8.5)	
Chronic kidney disease	40 (7.0)	32 (6.5)	8 (9.8)	
Liver cirrhosis	173 (30.2)	153 (31.2)	20 (24.4)	
Previous peptic ulcer	73 (12.8)	64 (13.1)	9 (11.0)	0.722
Antithrombotic agents	136 (23.8)	116 (23.7)	20 (24.4)	0.889
Aspirin	100 (17.5)	84 (17.1)	16 (19.5)	
Clopidogrel	42 (7.3)	36 (7.3)	6 (7.3)	
Dual antiplatelet therapy	25 (4.4)	21 (4.3)	4 (4.9)	
Warfarin	12 (2.1)	11 (2.2)	1 (1.2)	
DOAC	16 (2.8)	15 (3.1)	1 (1.2)	
NSAID use	42 (7.3)	36 (7.3)	6 (7.3)	0.992
**Presenting symptoms**				0.700
Hematemesis	297 (51.9)	258 (52.7)	39 (47.6)	
Melena	233 (40.7)	196 (40.0)	37 (45.1)	
Hematochezia	42 (7.3)	36 (7.3)	6 (7.3)	
SBP, mmHg	115 (51–226)	114 (51–226)	122 (67–194)	0.015
Heart rate, beats/min	97 (35–165)	98 (35–165)	97 (50–149)	0.806
**Laboratory findings**				
Hemoglobin, g/dL	8.9 (2.6–19.1)	9.1 (2.6–14.7)	8.8 (3.2–19.1)	0.339
BUN/Cr ratio > 30	303 (53.0)	256 (52.2)	47 (57.3)	0.406
Platelet, × 10^3^/µL	195 (8–801)	196 (8–801)	185 (22–440)	0.676
Prothrombin time, %	82 (4–137)	81 (4–127)	90 (8–137)	0.008
Time to endoscopy, hour	2.4 (0.4–45.0)	2.2 (0.4–5.9)	9.9 (6.0–45.0)	< 0.001
Glasgow-Blatchford score	11 (0–18)	11 (0–18)	10 (0–16)	0.334

Data are shown as median (range) or number (%).

*BUN/Cr ratio* ratio of blood urea nitrogen to creatinine, *DOAC* direct oral anticoagulant, *NSAID* non-steroidal anti-inflammatory drug, *SBP* systolic blood pressure.

Regarding the presenting symptoms, 51.9% of the patients had hematemesis, and 40.7% had melena. When comparing the urgent and elective endoscopy group, the median systolic blood pressure was significantly lower in the urgent group (*p* = 0.015). The GBS was 11 in the urgent group and 10 in the elective group, respectively (*p* = 0.334). The proportion of patients with GBS greater than 7 was 75.1% (368/490) in the urgent group and 67.1% (55/82) in the elective group (*p* = 0.135).

### Endoscopic findings and clinical outcomes

The median time to endoscopy was 2.4 h (range, 0.4–45.0 h). Endoscopic diagnoses included peptic ulcer (n = 310, 54.2%), gastroesophageal varix (n = 142, 24.8%), Mallory-Weiss syndrome (n = 49, 8.6%), and malignancy (n = 31, 5.4%) (Table 2). Endoscopic treatment was performed in 338 (59.1%) patients, which was successful in 334 patients. Two patients with duodenal ulcers required trans-arterial embolization after failed endoscopic treatment. One patient who had a gastric gastrointestinal stromal tumor underwent surgery, and the remaining patient with a duodenal ulcer had conservative management after confirmation of the absence of contrast extravasation on computed tomography scan with angiography. In one patient, duodenal varix was found at endoscopic examination and embolization was performed immediately as the first-line treatment.

**Table 2:** Endoscopic findings and clinical outcomes.

	Total (N = 572)	Urgent (n = 490)	Elective (n = 82)	*P* value
**Diagnosis**				0.569
Peptic ulcer	310 (54.2)	260 (53.1)	50 (61.0)	
Malignancy	31 (5.4)	27 (5.5)	4 (4.9)	
Mallory-Weiss syndrome	49 (8.6)	46 (9.4)	3 (3.7)	
Gastroesophageal varix	142 (24.8)	124 (25.3)	18 (22.0)	
Others^a^	40 (7.0)	33 (6.7)	7 (8.5)	
**Endoscopic treatment**	338 (59.1)	297 (60.0)	41 (50.0)	0.089
Thermocoagulation	170 (29.7)	147 (30.0)	23 (28.0)	
Hemoclip	22 (3.8)	21 (4.3)	1 (1.2)	
Injection	31 (5.4)	29 (5.9)	2 (2.4)	
Band ligation	139 (24.3)	122 (24.9)	17 (20.7)	
Combination	42 (7.3)	37 (7.6)	5 (6.1)	
Embolization	3 (0.5)	3 (0.6)	0	1.000
RBC transfusion, unit	2 (0–16)	2 (0–16)	2 (0–8)	0.346
Rebleeding (n = 571)	34 (6.0)	29 (5.9)	5 (6.1)	1.000
Mortality	31 (5.4)	26 (5.3)	5 (6.1)	0.791
Hospital stay, days	6 (1–128)	6 (1–128)	6 (1–86)	0.401

^a^Others include marginal ulcer, acute gastric mucosal lesion, angioectasia, tuberculosis, esophageal ulcer, jejunal ulcer, gastrointestinal stromal tumor, and neuroendocrine tumor.

**Figure 2:**
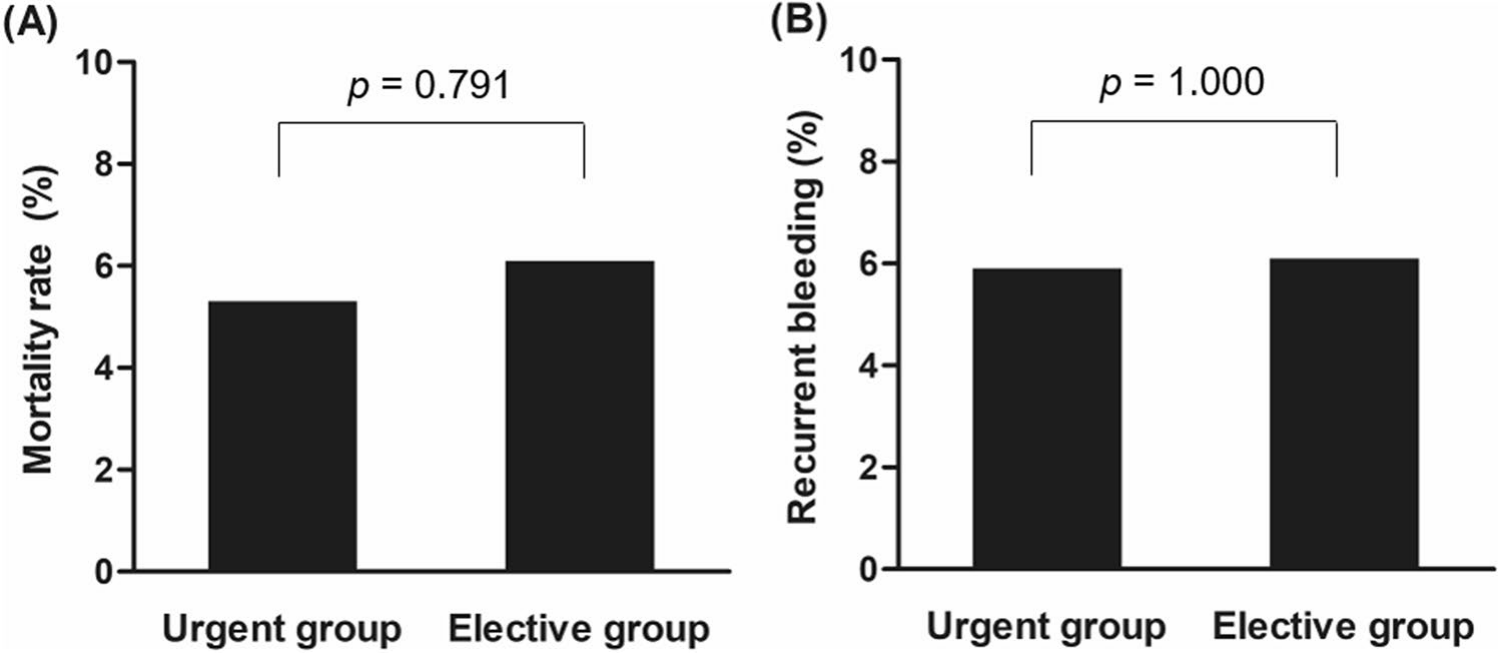
Mortality and recurrent bleeding rates within 30 days. (**A**) The mortality rate did not differ between the urgent (≤ 6 h from presentation) and elective (> 6 h from presentation) endoscopy groups, (**B**) the recurrent bleeding rate did not differ between the groups.

The mortality rate within 30 days was 5.4% in total (31/572), 5.3% (26/490) in the urgent endoscopy group and 6.1% (5/82) in the elective endoscopy group, respectively (*p* = 0.791) (Fig. 2). In a subgroup analysis of patients with variceal bleeding (n = 142), mortality rate did not differ between the two groups (Supplementary Table 1). In-hospital death occurred in 23 patients; the cause of death was hypovolemic shock associated with uncontrolled bleeding in 4 patients, and hepatic failure in 12 patients.

The rebleeding rate was assessed in 571 patients, excluding the patient who underwent embolization immediately after endoscopy. The rebleeding rate within 30 days was 6.0% (34/571) in total, 5.9% (29/489) in the urgent group, and 6.1% (5/82) in the elective group (*p* = 1.000). The median duration of hospital stay and the amount of transfused RBC did not differ between two groups.

Among the various clinical characteristics, age and amount of RBC transfusion were associated with mortality in univariate analysis (Table 3). Multivariate analysis showed that age (OR 1.038, 95% CI 1.008–1.069, *p* = 0.013) and RBC transfusion (OR 1.384, 95% CI 1.178–1.627, *p* < 0.001) were associated with mortality.

**Table 3:** Factors associated with mortality.

	Univariate analysis			Multivariate analysis		
	OR	95% CI	*P* value	OR	95% CI	*P* value
Age	1.035	1.006–1.064	0.017	1.038	1.008–1.069	0.013
Comorbidity	3.659	0.860–15.569	0.079			
Variceal bleeding	1.997	0.944–4.223	0.070			
Glasgow-Blatchford score	1.115	0.999–1.243	0.051	1.045	0.926–1.180	0.476
RBC transfusion	1.391	1.194–1.621	< 0.001	1.384	1.178–1.627	< 0.001
Urgent endoscopy	0.863	0.322–2.335	0.863			

*BUN/Cr ratio* ratio of blood urea nitrogen to creatinine, *CI* confidence interval, *OR* odds ratio, *RBC* red blood cell.

## Discussion

In this study, we compared the clinical outcomes of 572 patients with acute UGIB by the timing of emergency endoscopy. The mortality rate within 30 days and the recurrent bleeding rate did not differ between the patients who underwent urgent endoscopy within 6 h of presentation and those examined later. Logistic regression analysis also showed that urgent endoscopy was not associated with lower mortality rate, while age and the amount of transfusion were associated with increased mortality.

Most guidelines recommend performing endoscopy within 24 h of presentation among patients with acute UGIB^3–6^. Endoscopy performed within 24 h was associated with a reduced length of hospital stay, and delayed endoscopy was associated with higher mortality in patients with UGIB^8,15^. However, previous studies have shown conflicting results as to whether an earlier endoscopy within 6 to 12 h after presentation can offer more benefit for patients with UGIB. A retrospective study of 934 high-risk patients with GBS ≥ 12 found that endoscopy performed within 13 h resulted in a lower mortality rate and shorter hospital stays than later endoscopy^9^. In addition, a cohort study showed that urgent endoscopy within 6 h was an independent predictor of a lower mortality rate compared with elective endoscopy (6–48 h) in high-risk patients with GBS > 7^10^.

In contrast to these results, a retrospective study of 169 patients with acute non-variceal UGIB revealed no significant difference in mortality rate, rebleeding rate, or hospital stay between patients receiving endoscopy within 6 h and within 6–24 h^16^. Similarly, another study showed that early endoscopy within 12 h was not associated with reduced mortality compared with later endoscopy within 12–24 h^17^. In a recent randomized controlled trial, a total of 516 high-risk patients (GBS ≥ 12) with overt signs of acute UGIB, including variceal bleeding, were randomized to urgent (within 6 h after gastroenterology consultation) and early (within 24 h) endoscopy groups^7^. Of note, some patients were not treated as assigned because of hemodynamic instability or changes in their medical conditions. There was no significant difference between the urgent and early groups in mortality rate (8.9% vs 6.6%, hazard ratio, 1.35; 95% CI 0.72–2.54) and rebleeding rate (10.9% vs 7.8%, hazard ratio, 1.46; 95% CI 0.83–2.58). In addition, another recent retrospective study of 6474 patients presented with acute UGIB showed that urgent endoscopy (within 6 h) showed worse outcomes compared with early (between 6 and 24 h) and late (between 24 and 48 h) endoscopy groups^18^. In the present study, we included patients with acute UGIB regardless of the final diagnosis. The 30-day mortality rate and rebleeding rates did not differ between the patients who underwent endoscopy within 6 h and those who underwent endoscopy after 6 h. These results support recent guidelines that recommends performing endoscopy within 24 h following hemodynamic resuscitation in patients with UGIB and that do not encourage emergent (within 12 h) endoscopy^4,5^.

Endoscopy performed early in the clinical course is considered useful to triage patients based on the endoscopic findings. Patients with a low risk of recurrent bleeding could be discharged earlier. In addition, early intervention within 24 h was associated with a shorter length of hospital stay^19^. In contrast, some studies have suggested that earlier endoscopy may yield more high-risk endoscopic stigmata that would have been resolved with proton pump inhibitor therapy. A previous study showed that more retained blood and more actively bleeding lesions were found in patients who underwent endoscopic examination within 8 h from presentation compared with those who underwent endoscopy between 8 and 24 h^8^. A recent randomized trial also showed that patients with active bleeding or visible vessels and those who required endoscopic treatment were more frequently found in the urgent endoscopy (within 6 h after gastroenterology consultation) group than in the early endoscopy (within 24 h) group^7^. In the present study, we found that the proportion of patients who underwent endoscopic treatment was higher in the urgent group than in the elective group, and the need for endoscopic treatment was not associated with reduced recurrent bleeding. These results suggest that initial hemodynamic resuscitation and proton pump inhibitor therapy are more beneficial than earlier endoscopy within 6 h for improving clinical outcomes^20^.

Several factors have been reported to be associated with mortality in patients with UGIB, including comorbidities, vital signs, and failed endoscopic treatment^10,21–24^. In the present study, the clinical factors associated with mortality were age and RBC transfusion. A previous observational study that investigated 186 patients in Korea also showed that age (≥ 65 years) was an independent predictive factor for mortality^21^. Worse outcomes in the elderly might be attributable to a tendency to having multiple comorbidities and the susceptibility to physiological changes of elderly patients. There were also discrepancies regarding the impact of RBC transfusion on mortality in previous studies^21,22,24^. This may be due to the demographics of the patients and the study design, and additional studies considering various demographic characteristics are necessary to identify clinical factors affecting outcomes of patients with acute UGIB.

There are several limitations of this study. First, this is a retrospective study. Although it is desirable to design randomized control trial, performing such a study would be difficult and may have ethical issue since a deliberate delay in endoscopic procedures may lead to fatal outcomes. Future prospective studies would be beneficial to confirm the limited role of urgent endoscopy. Second, because this is a single-center study, the results may not represent the overall national demographics. Indeed, in our center, most endoscopic examinations were performed within 6 h, and this clinical practice pattern may influence the lack of differences in mortality rates between groups. Since the resources required to perform emergency procedures are limited, the results of this study cannot be readily generalized. Another possible limitation of this study is that our study population consists of heterogeneous patients, including those with gastroesophageal variceal bleeding. However, in clinical practice, it is often challenging to discriminate patients with variceal bleeding from non-variceal bleeding at the time of presentation, even after a thorough history taking. Since the decision whether to perform endoscopy immediately or not is made based on the information obtained at presentation, it is reasonable to include all patients with UGIB, including variceal bleeding, to determine the role of urgent endoscopy in a real-world setting.

In conclusion, urgent endoscopy within 6 h was not significantly associated with lower mortality and rebleeding rates. We also found that age and RBC transfusion were the factors related to mortality. Based on these findings, we suggest performing elective endoscopy after hemodynamic stabilization in patients with suspected acute UGIB.

## Supplementary Information

Supplementary Table 1.
