# Supplementary material for: Timing of endoscopy in patients with upper gastrointestinal bleeding
Source: Sci Rep. 2022 Apr 27;12:6833. doi: 10.1038/s41598-022-10897-3 (PMC9046398; doi:10.1038/s41598-022-10897-3)
Supplement: Supplementary file 1 — Supplementary Table 1. [file 41598_2022_10897_MOESM1_ESM.docx]

**Supplementary Table 1** Characteristics and clinical outcomes of patients with variceal bleeding.

|  | Total  (n = 142) | Urgent  (n = 124) | Elective  (n = 18) | *P* value |
| --- | --- | --- | --- | --- |
| Age, years | 57 (29–83) | 57 (29–83) | 60 (43–75) | 0.916 |
| Male | 107 (75.4) | 91 (73.4) | 16 (88.9) | 0.241 |
| Presenting symptoms |  |  |  | 0.250 |
| Hematemesis | 112 (78.9) | 100 (80.6) | 12 (66.7) |  |
| Melena | 26 (18.3) | 21 (16.9) | 5 (27.8) |  |
| Hematochezia | 4 (2.8) | 3 (2.4) | 1 (5.6) |  |
| SBP, mmHg | 114 (51–169) | 114 (51–169) | 111 (67–163) | 0.890 |
| Heart rate, beats/min | 100 (50–160) | 102 (56–160) | 91 (50–145) | 0.335 |
| Glasgow-Blatchford score | 11 (3–17) | 11 (3–17) | 11 (4–15) | 0.827 |
| Endoscopic treatment | 132 (93.0) | 116 (93.5) | 16 (88.9) | 0.615 |
| RBC transfusion | 2 (0–11) | 2 (0–11) | 2 (0–8) | 0.663 |
| Rebleeding | 10 (7.0) | 8 (6.5) | 2 (11.1) | 0.615 |
| Mortality <30 days | 12 (8.5) | 12 (9.7) | 0 | 0.363 |
